# Electronic field protocols for prehospital care quality improvement in Lithuania: a randomized simulation-based study

**DOI:** 10.1186/s13049-023-01150-5

**Published:** 2023-11-21

**Authors:** Ieva Paliokaite, Zilvinas Dambrauskas, Paulius Dobozinskas, Evelina Pukenyte, Aida Mankute-Use, Dinas Vaitkaitis

**Affiliations:** 1 Department of Emergency Medicine, Lithuanian University of Health Sciences, Kaunas, Lithuania; 2 Department of Surgery, Lithuanian University of Health Sciences, Kaunas, Lithuania; 3 Department of Disaster Medicine, Lithuanian University of Health Sciences, Kaunas, Lithuania; 4 Department of Infectious Diseases, Lithuanian University of Health Sciences, Kaunas, Lithuania

**Keywords:** Prehospital, Electronic field protocols, Cognitive aids, Clinical decision support, Simulation

## Abstract

**Background:**

Prehospital emergency care is complex and influenced by various factors, leading to the need for decision-support tools. Studies suggest that cognitive aids improve provider performance and patient outcomes in clinical emergencies. Electronic cognitive aids have rarely been investigated in prehospital care. Therefore, this study aimed to evaluate the effects of the electronic field protocol (eFP) module on performance, adherence to the standard of care, and satisfaction of prehospital care providers in a simulated environment.

**Methods:**

This randomised simulation-based study was conducted at the Lithuanian University of Health Sciences in Kaunas, Lithuania. The simulation scenarios were developed to test 12 eFPs: adult resuscitation, pediatric resuscitation, delivery and postpartum care, seizures in pregnancy, stroke, anaphylaxis, acute chest pain, acute abdominal pain, respiratory distress in children, severe trauma, severe infection and sepsis, and initial neonatal evaluation and resuscitation. Sixteen prehospital practitioners with at least 3 years of clinical experience were randomly assigned to either use the eFP module or perform without it in each of the 12 simulated scenarios. Participant scores and adherence to standardised checklists were compared between the two performance modes. Participant satisfaction was measured through a post-simulation survey.

**Results:**

A total of 190 simulation sessions were conducted. Compared to the use of memory alone, the use of the eFP module significantly improved participants’ performance in 10 out of the 12 simulation scenarios. Adherence to the standardised checklist increased from 60 to 85% (*p* < 0.001). Post-simulation survey results indicate that participants found the eFP module easy to use and relevant to prehospital clinical practice.

**Conclusions:**

The study findings suggest that the eFP module as a cognitive aid can enhance prehospital practitioners’ performance and adherence to the standard of care in simulated scenarios. These results highlight the potential of standardised eFPs as a quality improvement step in prehospital care in Lithuania.

**Supplementary Information:**

The online version contains supplementary material available at 10.1186/s13049-023-01150-5.

## Background

Emergency medical services (EMS) providers are expected to be able to rapidly recognise and manage a wide variety of conditions, determine the acuity level, and make appropriate decisions regarding transportation to further treatment facilities. Prehospital decision-making is challenging and depends on system influences, environmental factors, patient characteristics, available resources, and the provider’s experience and knowledge [1]. However, relying solely on professional training and experience leaves room for medical errors and adverse events. Therefore, the demand for guidelines, protocols, and other decision-support tools is increasing in prehospital healthcare [2–6].

Despite the significant cognitive overload, EMS providers must adhere to current guidelines and recommendations to ensure optimal patient outcomes. However, adherence is influenced by many factors, such as perceptions of guideline quality, provider experience, risk tolerance, patient and organisational circumstances, and implementation issues [2, 3, 7]. Ebben et al. [7] argued that guidelines contain too many recommendations for providers to adhere to while making critical choices. Thus, there is a growing need for more efficient and practical forms of decision support.

Cognitive aids (CAs) such as visual aids, checklists, mnemonics, and flowcharts are described as tools to support cognitive processes during complex and demanding tasks [8]. Designed to address cognitive challenges, these aids are used in real time [9]. Their purpose is to enhance patient safety, improve health management efficiency, and deliver better patient outcomes, all in combination with the provider’s clinical judgment and training [10]. A recent systematic review and meta-analysis demonstrated the positive impact of CAs on reducing errors, increasing the rate of correctly performed steps, and improving teamwork in clinical emergencies [11]. However, the majority of research on CAs has focused on in-hospital settings [12–19], with limited literature available specifically for prehospital care [20–24].

For a few decades, prehospital practitioners have used diagnostic and treatment field protocols to reduce errors and enhance their performance [25, 26]. Nevertheless, considerable variation exists in the content and usage practices of these protocols across different countries and regions [7, 27]. Even in regions where field protocols are mandatory, prehospital providers apply them retrospectively, placing greater emphasis on their knowledge base [1]. In a study by Hagiwara et al. [28], the paper format of the guidelines or protocols was identified as a major obstacle to their effective use by prehospital practitioners, leading to the creation of various homemade formats. The authors proposed electronic guidelines connected to patient care records and accessible during patient transport as a decision-support solution. A well-designed CA should offer clear and goal-specific guidance to help providers prioritise actions, recall treatment steps, consider relevant diagnoses, and deliver evidence-based care [10]. However, little is known about the development of such systems in prehospital care.

There are wide disparities in prehospital services in Lithuania. Currently, EMS lack evidence-based field protocols, manuals, and other CA systems for practitioners that are approved nationwide. Moreover, standards of care vary between institutions, with some implementing protocols approved by the administration while others operating without such regulations. However, the exact usage habits among EMS providers in these institutions remain unknown. Methodological recommendations for prehospital care providers based on international guidelines and approved by the Ministry of Health are often impractical in everyday practice and therefore used more as educational material. At the scene, prehospital providers have limited decision support and rely primarily on online medical consultations. Consultations are provided by the chief medical doctor in the dispatch centre upon the request of EMS teams. Consultations with neurologists or cardiac intensive care units regarding stroke and STEMI patients are also available since the EMS providers must inform receiving hospitals about these patients [29, 30]. Nevertheless, there is still a lack of decision support in other clinical situations. The prehospital patient care record (PCR) form approved by the Ministry of Health was implemented in 2014 [31]. However, it serves mainly as a storage system with little capability to support decision-making (e.g., stroke, trauma activation criteria).

Standardised interactive field protocols with evidence-based diagnostic and management steps for EMS providers integrated with PCR and allowing for a quick review of critical information at any time, especially on route to the patient, are needed. In this study we used simulation scenarios to assess the impact of the CA provided to EMS practitioners before departure to the scene. The aim of this study was to evaluate the effects of a newly developed electronic field protocol (eFP) module as a CA tool on prehospital providers’ adherence to the standard of care, performance outcomes, and overall satisfaction in a simulated environment.

## Methods

### Study design and participants

We conducted this prospective, randomised, unblinded study at the Lithuanian University of Health Sciences (LUHS) between 17 October 2022 and 18 November 2022. The study was approved by Kaunas Regional Biomedical Research Ethics Committee (protocol no. BE-2-80). The study was conducted in accordance with the Declaration of Helsinki’s ethics standards and its latest amendments. All subjects provided informed consent for inclusion before participating in this study with the possibility to withdraw anytime. The manuscript complies with the Simulation-Based Research Extention for the CONSORT Statement [32].

Prehospital care practitioners from Kaunas Emergency Medical Service Station in Lithuania were invited to participate in simulation-based testing of the eFP module. We enrolled 16 volunteer practitioners (paramedics, community nurses, and emergency care nurses) with at least 3 years of work experience in EMS to ensure a similar level of competence and to avoid the effect of novice providers learning from the protocols rather than using them as a CA. We aimed to have a subject group as homogenous as possible, so we excluded medical doctors. They have different knowledge backgrounds compared to paramedics or nurses and are not the predominant personnel providing prehospital care nationwide. The primary effect variable used for power calculation was the difference in overall simulation performance when using the eFP module compared with the conventional group. With the assumption of a difference in means of 20%, a standard deviation of 10%, a statistical power of 0.8 and a risk of 0.05 for type-1 error, total sample size of 10 was required.

### Field protocols and simulation scenarios

**Fig. 1:**
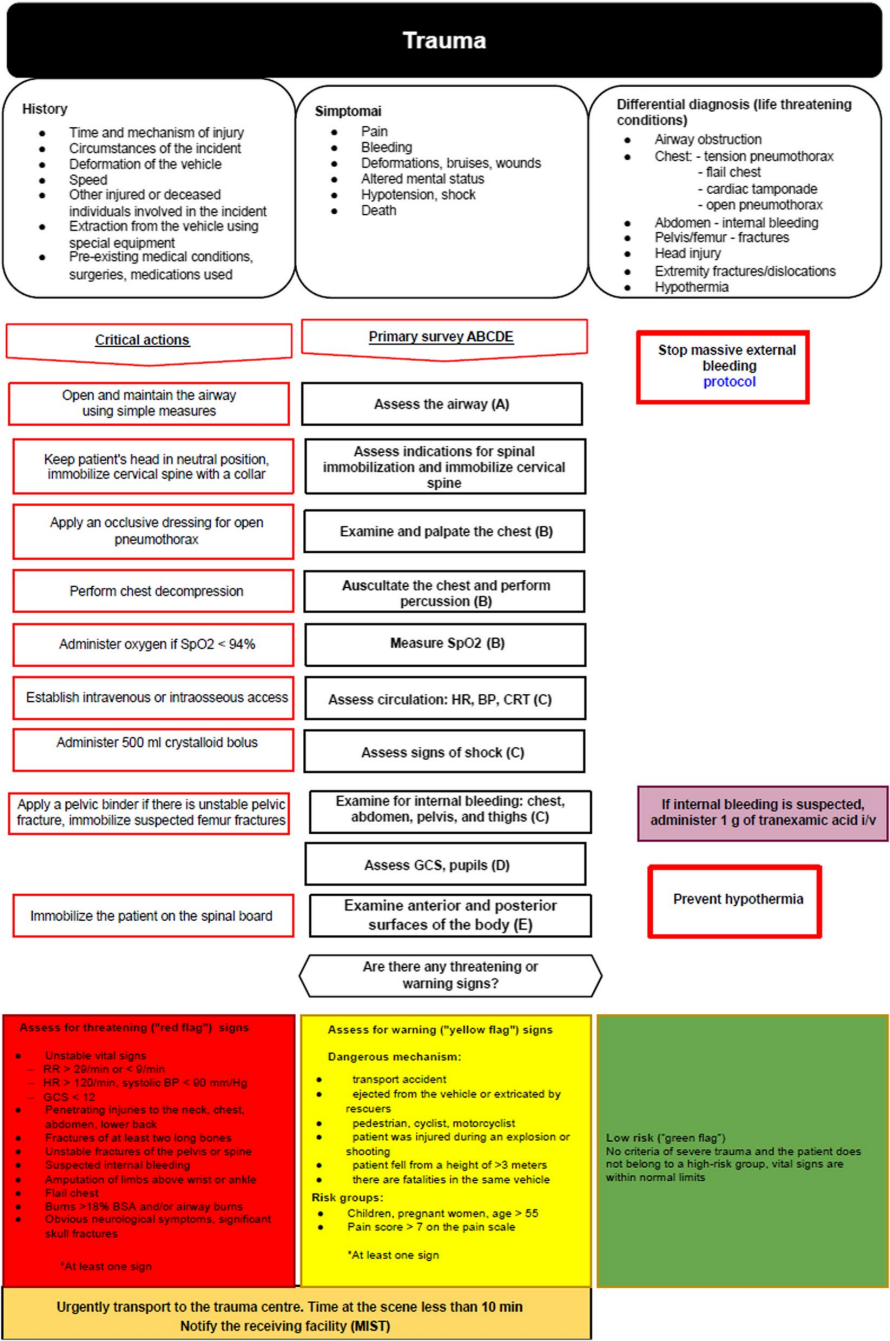
Field protocol example

We tested a set of 12 diagnostic and treatment field protocols newly developed for this study by interdisciplinary LUHS expert groups. Study participants were not familiar with the protocols before the simulations. These protocols were developed as standardised CAs for prehospital practitioners based on current guidelines and recommended practices in each field, as well as national healthcare system regulations and available ambulance resources. The Delphi methodology was used for the development process. Field protocols covered the following 12 prehospital care topics listed in Table 1. The protocols were designed as standardised information sheets (Fig. 1) with critical diagnostic, treatment, and decision-making steps; medication-dose and/or equipment-selection calculators where appropriate; and the most important information about complex, dangerous, or rare clinical conditions that could be reviewed within a few minutes.

**Table 1:** The list of field protocol topics

	Topics of field protocols
1	Adult resuscitation
2	Pediatric resuscitation
3	Delivery and postpartum care
4	Seizures in pregnancy
5	Stroke
6	Anaphylaxis
7	Acute chest pain
8	Acute abdominal pain
9	Respiratory distress in children
10	Severe trauma
11	Severe infection and sepsis
12	Initial neonatal evaluation and resuscitation

Simulation material for this study was created by experienced LUHS instructors. Scenarios and standardised evaluation checklists were developed for each of the 12 field protocols based on the same guidelines and standard of care recommendations. The authors assessed the checklists for usability during trial runs in the simulation centre. Checklist point values were binary: 0 = not performed and 1 = performed. The total checklist score for each field protocol topic was different.

Before the study, all field protocols were transferred into an electronic platform (HybridLab® at LUHS), and the electronic field protocol module was created for testing. The electronic platform allowed for the interactive eFP design with links from the main information sheet to calculators or other protocols. The HybridLab® platform was also used to contain simulation material, perform assessments of participant performance, and collect performance data during simulation sessions.

Before the study, all participants were informed that 12 diagnostic and treatment field protocols would be tested and were briefed on using all elements of the eFP module. Each subject was expected to participate in 12 sessions of predetermined simulated clinical scenarios with or without the assistance of the eFP module. Before simulation sessions, the randomization of eFP availability for each field protocol topic was performed with a research randomizer (www.randomizer.org). Until the beginning of the simulation session, participants did not know which protocol they would be assigned to simulate or whether they would have to work with or without the assistance of the eFP module.

All simulation sessions were managed and scored by a consistent group of study researchers who were previously involved in developing the scenarios and evaluation checklists. Simulations were conducted at the LUHS Simulation Centre using appropriate manikins for specific scenarios, real ambulance equipment and medications, and standard Lithuanian EMS electronic PCR documentation to maintain high-fidelity conditions as much as possible.

The time limit for the simulation sessions was 30 min. Before each simulation, the participant received a brief synopsis of the case, similar to the information provided by a dispatcher during an emergency call. Scenarios with or without the assistance of the eFP module were identical. Participants allocated to work without assistance had 15 min to plan their actions independently, while those working with the eFP module spent 15 min preparing their plan according to the suggested protocol steps. Depending on the field protocol topic, possible areas of suggestion were the critical steps of patient examination and treatment, the most commonly used medications and their doses, the most commonly used medical equipment and its application, differential diagnosis list, and recommendations on transportation to the appropriate treatment facility. Standard prehospital PCR documentation was used in all simulations.

Two researchers worked with the participant during each simulation session: the first researcher led the simulation scenario and assessed participants’ performance in real time using a predetermined electronic standardised checklist (assessor), while the second assumed the role of the EMS team member (assistant) and carried out the instructions of the participating EMS practitioner (team leader). EMS practitioners acted independently during the simulation, with the assisting researcher performing only auxiliary technical actions. The assisting researcher could not provide any suggestions regarding diagnostic and treatment decisions, clarify the simulation scenario, or otherwise influence the participant’s decisions and/or actions. Scripted responses were provided for the participants by the assessor about the present condition of the simulated patient. After the simulation, the researchers provided the participant with brief feedback.

### Post-simulation survey

After the simulations, all participants were asked to complete an anonymous survey. The survey comprised 36 statements regarding their perceptions of the usefulness of the eFP module and the simulations. A Likert scale ranging from 1 (*very inaccurate*) to 10 (*very accurate*) was used to evaluate the degree of the participant’s agreement with the statement. The survey included a section for free-text comments.

### Statistical analysis

After the study was complete, data were deidentified and exported from the HybridLab® platform for analysis. IBM SPSS Statistics 27.0 software was used for statistical analysis. Checklist scores were tallied for every simulation, and the number of simulation sessions per participant and per performance method was calculated. Results of individual performances are demonstrated as the number of simulations per participant and evaluation scores from checklists. Means and standard deviations (SD) for checklist scores were calculated for both performance modes (with and without assistance) of each protocol, and the means were compared using Student’s *t* test. In both performance modes, the percentage adherence to the checklists was also determined. All reported *p* values are two-sided with statistically significant differences when *p* < 0.05.

## Results

### Analysis of participants’ performance

We conducted a total of 190 simulation sessions: 95 sessions with and 95 without the assistance of the eFP module. Fourteen participants performed all 12 field protocol simulation sessions. One participant did not attend the simulation of the “Acute chest pain” protocol, and one participant of the “Initial neonatal evaluation and resuscitation” protocol. Participants attended four to seven sessions with assistance and five to seven sessions without assistance. The detailed characteristics of all participants’ performance in different simulation scenarios and their individual scores as checklist points are presented in Additional file 1. Mean [SD] performance scores were higher with the assistance of the eFP module compared to the conventional group (30.43 [8.39] vs. 21.7 [8.06], *p* = 0.016, respectively). Performance with the eFP module was significantly better in each field protocol scenario subgroup, except for the “Pediatric resuscitation” and “Delivery and postpartum care” protocols (see Table 2).

**Table 2:** Comparison of mean checklist scores for every protocol simulation with and without the assistance of the eFP module

	Protocol title and performance mode	Number of simulations	Mean checklist score	SD	*p*
1	Adult resuscitation (without assistance)	8	36.00	8.84	0.021
	Adult resuscitation (with assistance)	8	44.62	3.20	
2	Pediatric resuscitation (without assistance)	7	33.42	9.34	0.058^a^
	Pediatric resuscitation (with assistance)	9	43.00	9.09	
3	Delivery and postpartum care (without assistance)	9	16.00	6.48	0.078^a^
	Delivery and postpartum care (with assistance)	7	21.42	4.35	
4	Seizures in pregnancy (without assistance)	9	7.77	4.26	< 0.001
	Seizures in pregnancy (with assistance)	7	18.57	1.81	
5	Stroke (without assistance)	7	26.14	5.58	0.007
	Stroke (with assistance)	9	35.00	5.56	
6	Anaphylaxis (without assistance)	7	18.57	3.99	0.002
	Anaphylaxis (with assistance)	9	24.44	2.29	
7	Acute chest pain (without assistance)	6	26.00	6.32	0.034
	Acute chest pain (with assistance)	9	31.55	2.69	
8	Acute abdominal pain (without assistance)	9	19.44	6.98	0.001
	Acute abdominal pain (with assistance)	7	31.57	4.27	
9	Respiratory distress in children (without assistance)	7	13.42	5.02	0.008
	Respiratory distress in children (with assistance)	9	19.33	2.44	
10	Severe trauma (without assistance)	9	23.66	4.84	0.002
	Severe trauma (with assistance)	7	32.42	4.39	
11	Severe infection and sepsis (without assistance)	9	17.11	7.83	0.001
	Severe infection and sepsis (with assistance)	7	30.00	4.08	
12	Initial neonatal evaluation and resuscitation (without assistance)	8	22.87	5.48	< 0.001
	Initial neonatal evaluation and resuscitation (with assistance)	7	33.28	1.25	

^a^No statistical significance

### Adherence to the standard of care

**Fig. 2:**
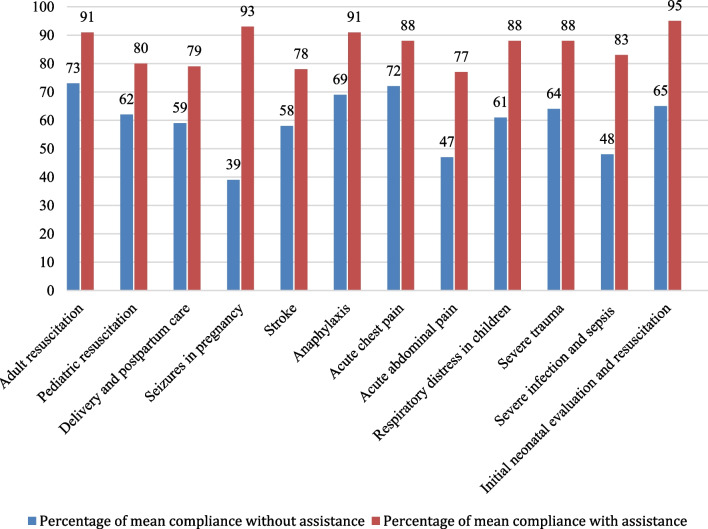
Participants’ adherence to the standardised checklists in simulations with and without the assistance of the eFP module

Using the eFP module significantly increased average adherence with the standardised checklist—from 60 to 85% (*p* < 0.001). Participants achieved higher mean adherence with the checklists than our intended 75% in all simulated field protocols with the assistance, and in no case was this adherence achieved without the assistance of the eFP module (see Fig. 2).

### Post-simulation survey results

All participants completed the post-simulation survey. Respondents gave positive ratings (8–10 on the Likert scale) for most questionnaire items regarding the simplicity of the material, design of the CA, relevance of the eFP in the clinical setting, and their self-confidence and satisfaction when using the tool. Participants chose the most negative ratings (1–3 on the scale) for questionnaire statements that mentioned too much information, irrelevant contents, difficulties in understanding the material of protocols or the user’s interface of the eFP module. The results show that the material of the eFP module was easy to use, compelling in design, understandable, and relevant to clinical practice. Moreover, the results indicate that this tool will help providers feel better prepared to care for the patients. Participants found the simulations useful, felt positive after the sessions, and appreciated the feedback. Results of the questionnaire and representative comments of the participants are presented in Additional file 2.

## Discussion

We aimed to evaluate the effect of the eFP on EMS providers’ performance and adherence to the standard of care in managing simulated scenarios compared with their performance based on memory alone. The study demonstrated that the assistance of the eFP module significantly positively impacted participants’ performance during 10 out of the 12 predetermined field protocol simulation scenarios. Furthermore, the percentage mean adherence to the standardised checklist was significantly higher when participants used the eFP module. Similarly, previous studies in prehospital [20, 22–24] and hospital settings [15, 18, 33, 34] have shown improvement in provider performance and adherence to the guidelines using different types of CAs and decision support systems. However, some studies show conflicting results. For example, McMillan et al. found that CAs did not prompt the initiation of cardiopulmonary resuscitation, which resulted in errors during simulated paediatric cardiac arrest situations [17]. The authors discussed that the reason was an incorrect choice of the CA, possibly due to inadequate training and experience of participants or flaws in the CA design. Problem-oriented eFPs used in our study could prevent participants from making incorrect choices. Our study did not show such negative effects as mentioned above.

Nevertheless, we found no statistical difference in participant performance in simulations of “Pediatric resuscitation” and “Delivery and postpartum care” topics. We can only speculate that participants’ experience with these situations in real life and background training might have superseded the eFP suggestions and resulted in similar actions with or without the eFP during a given scenario. This finding also suggests a failure of the particular eFPs to trigger the appropriate tactics. Another possible reason could be a lack of simulation fidelity in these topics. Small number of participants could also influence the statistical significance as there were also small number of simulations performed.

The difference in the standard deviation between the group that utilized the CA and the group without assistance can be noticed. Lower standard deviations in the group with assistance across all scenario topics suggest a trend towards more stable performance and more consistent adherence to recommended protocols.

Our study participants had at least 3 years of experience in EMS, yet despite the assistance of the eFP module, most of them did not achieve the highest evaluation scores and adherence with the standardised checklists. The reason could be that the participants were exposed to the full eFP material for the first time only 15 min before the simulation. This factor suggests that participants’ ability to retain prompt information may vary and could be influenced by the design and visualisation of the eFP module. Previous studies have shown that familiarisation with CAs [35] and a simple linear design rather than a branched design [36] are important factors for more effective CA use. The comparison of different eFP designs could be an area of future research. Trends in participants’ performance could be associated with different background knowledge compared to the eFP material. The function of CAs is not to have novices learn from them but to support providers in remembering critical information during cognitive crises [9]. Therefore, CAs may not trigger recall if providers’ background knowledge on the topic is different. However, CAs cannot supersede clinical expertise and decision-making and problem-solving abilities [33]. Nevertheless, our study demonstrates that the intended > 75% adherence with diagnostic and treatment protocol checklists using the eFP module was achieved in all scenarios.

Although this study involved a small sample of employees from a single institution, disparities in individual performance were observed among these employees, despite already inferior scores in simulation scenarios without the assistance of the eFP module. This finding highlights the importance of protocols and standardisation not only within a single EMS station but also across all institutions providing prehospital care in the country.

The post-simulation survey results indicate that participants found the material of the eFP module to be clear, concise, user-friendly, visually appealing, and relevant to their clinical practice, although some indicated that its complexity was too high. The eFP module was not intended for learning, as discussed earlier, yet some participant answers indicate this effect. Other studies show the positive perceptions of clinicians about the use of CAs, where participants indicated they would use these tools if the tools were available during an emergency [18, 37].

### Limitations

A limitation of this study is that it was performed in a simulation environment. Therefore, our findings should be interpreted within the context of our study design. The effect of the use of eFPs on clinical outcomes in the prehospital setting is yet to be established. Prehospital cases undoubtedly are more complex than we could simulate because of different and constantly changing environments, and whether the effect on adherence with recommendations would increase or decrease is unclear. The simulation environment, with the researchers present during the sessions, could also have impacted the results, as it could influence participants’ motivation to perform better. The wide range of scenario topics required different types of simulation resources, which negatively impacted simulation fidelity. Since some of the scenarios required scripted responses from the instructor and others could be conducted without them, we chose a unified method with the instructor (assessor) present. The second instructor could have provided answers on the progress of the scenario, but the concept of the EMS team member would have been lost. In situ simulation studies might better approximate the effectiveness of a new tool [18]. However, the feasibility of this type of study in a prehospital setting is limited.

Another limitation of this study is the small number of participants from a single urban EMS institution. Therefore, large-sample, multi-institutional studies need to be performed involving participants from different regions to investigate whether these results are generalizable. Furthermore, the study was conducted in the Lithuanian context, which can be limited in generalizability to other countries with different prehospital systems.

Another possible limitation is the risk of contamination because of information leakage among participants. The reason is that the study went on for a few weeks and the sample was a small group of participants from the same institution.

Subsequent studies should employ additional simulations to assess the participants’ performance using video surveillance without the presence of researchers. A more thorough analysis of the effectiveness of individual eFPs and continuous improvement of participant performance through consistent use of the eFP module should also be studied. Future research could also address the effects of the eFP module on teamwork and other non-technical skills, EMS providers’ performance in a real environment, and eFP integration with prehospital patient records.

## Conclusions

Our study shows that a standardised approach using the newly developed eFP module as a CA is effective in improving EMS providers’ performance and adherence to the standard of care in a simulated environment. This is a first step towards electronic decision support for prehospital providers and quality improvement in prehospital care in Lithuania.

### Supplementary Information

**Additional file 1**. Participants’ checklist scores in simulations.

**Additional file 2**. Participants’ perceptions of the electronic field protocol (eFP) module and simulation sessions.

## Data Availability

All data generated or analysed during this study are included in this published article and its additional files.

## Abbreviations

EMS: Emergency medical services
CA, CAs: Cognitive aid, cognitive aids
PCR: Patient care record
eFP: Electronic field protocol
LUHS: Lithuanian University of Health Sciences
